# Dr. Milton Bennett Titus

**Published:** 1916-12

**Authors:** 


**Dr. Milton Bennett Titus,** N. Y. Homoeopathic 1881, died in St. Luke's Hospital, Chicago, Oct. 14, from the effects of poison believed to have been self-administered. He was 58 years old. He was at one time President of the Allegany Co. Medical Society.

